# Supplementary material for: Spatial structure of disordered proteins dictates conductance and selectivity in nuclear pore complex mimics
Source: eLife. 2018 Feb 14;7:e31510. doi: 10.7554/eLife.31510 (PMC5826291; doi:10.7554/eLife.31510)
Supplement: Supplementary file 1. [file elife-31510-supp1.docx]

| **Protein** | **Hydrodynamic Diameter (nm)** |
| --- | --- |
| kap95 | 8.5 ± 0.8 |
| tCherry | 7.4 ± 1.4 |
| Nsp1 | 14.6 ± 2.1 |
| Nsp1-S | 13.5 ± 3.1 |
