# Supplementary material for: Spatial structure of disordered proteins dictates conductance and selectivity in nuclear pore complex mimics
Source: eLife. 2018 Feb 14;7:e31510. doi: 10.7554/eLife.31510 (PMC5826291; doi:10.7554/eLife.31510)
Supplement: Supplementary file 2. [file elife-31510-supp2.docx]

|  | **Nsp1** | **Nsp1-S** | **Nup98** | **Nsp1-head**  **(AA 1-172)** | **Nsp1-tail**  **(AA 173-637)** |
| --- | --- | --- | --- | --- | --- |
| **#aminoacid**  **(AA)** | 637 | 638 | 498 | 172 | 465 |
| **#charged AA (pos/neg)** | 135 | 135 | 25 | 5 | 130 |
| **#hydrophobic AA (F,I,L,V)** | 72 | 2 | 90 | 19 | 53 |
| **#hydrophobic AA (F,I,L,V,W,Y,A)** | 146 | 76 | 130 | 30 | 116 |
| **Stokes radius** $\boldsymbol{R}_{\mathbf{S}}$ **in nm (stand. dev.)** | 7.4 (0.6) | 8.4 (0.6) | 4.1 (0.3) | 3.2 (0.2) | 6.5 (0.5) |
| **Mass in Da** | 76440 | 76560 | 59760 | 20640 | 55800 |
| **Stokes volume,** **4π**$\boldsymbol{R}_{\mathbf{S}}^{\boldsymbol{3}}$**/3** **in ml** | 1.72E-18 | 2.44E-18 | 2.88E-19 | 1.37E-19 | 1.15E-18 |
| **Protein density:**  **mass per Stokes volume in mg/ml** | 73.9 | 52.1 | 344 | 250 | 80.9 |
